# Low-dose carbon monoxide inhalation to increase total hemoglobin mass and endurance performance: scientific evidence and implications

**DOI:** 10.3389/fphys.2024.1490205

**Published:** 2024-10-15

**Authors:** Hannes Gatterer, Tobias Dünnwald, Simon Woyke, Martin Faulhaber, Yorck Olaf Schumacher, Wolfgang Schobersberger

**Affiliations:** ^1^ Institute of Mountain Emergency Medicine, Eurac Research, Bolzano, Italy; ^2^ Institute for Sports Medicine, Alpine Medicine and Health Tourism (ISAG), UMIT TIROL-Private University for Health Sciences and Health Technology, Hall in Tirol, and University Hospital Innsbruck, Hall in Tirol, Austria; ^3^ Department of Anesthesiology and Intensive Care Medicine, Medical University of Innsbruck, Innsbruck, Austria; ^4^ Department of Sport Science, University of Innsbruck, Innsbruck, Austria; ^5^ Aspetar Orthopedic and Sports Medicine Hospital, Doha, Qatar

**Keywords:** endurance performance, carbon monoxide rebreathing, doping, hemoglobin mass, blood manipulation, altitude training

## Abstract

Recently, chronic intermittent inhalation of low-dose carbon monoxide (CO) has been postulated as a practice to increase total hemoglobin mass with potential beneficial effects on endurance performance. In this perspective article, we discuss the potential performance enhancing capabilities as well as the safety concerns, which include individual variability in CO response, and acute and chronic health effects. It is also important to note that according to the World-Anti-Doping-Agency (WADA), CO inhalation could fall under “*M1. Manipulation of Blood and Blood Components*“ and therefore could be considered a prohibited method if used as a non-diagnostic tool.

## 1 Introduction

Recent media reports have purported that professional cyclists are attempting to improve their exercise performance by inhaling carbon monoxide (CO) (https://escapecollective.com/exclusive-tour-riders-are-inhaling-carbon-monoxide-in-super-altitude-recipe/). Although this practice was never proven effective in elite athletes and is beyond any ethical discussion, it raises important questions about the performance enhancing and adverse effects of CO inhalation in competitive endurance sports.

CO is a molecule with a dual nature. While primarily known for its harmful effects at high doses (Smollin and Olson, 2010), research has revealed potential therapeutic (Bansal et al., 2024; Motterlini and Otterbein, 2010) and diagnostic applications (Schmidt and Prommer, 2005; Siebenmann et al., 2017; Zavorsky and Smoliga, 2017) for low-dose CO administration. At physiological levels and slightly elevated pharmacological concentrations, CO plays crucial roles in the body, including modulating inflammation and oxidative stress, as well as regulating mitochondrial biogenesis and angiogenesis (Bansal et al., 2024; Motterlini and Otterbein, 2010). Moreover, CO administration (mainly by CO rebreathing) was reported to directly increase erythropoietin concentration (Montero and Lundby, 2019) with potential effects on red blood cell mass and exercise performance. In research and clinical practice, low-dose CO is utilized as a tracer to assess lung diffusion capacity and total hemoglobin mass (tHb-mass) (Schmidt and Prommer, 2005; Siebenmann et al., 2017; Zavorsky and Smoliga, 2017; Amann, 2012; Robach et al., 2012; Nummela et al., 2021; Saunders et al., 2013).

## 2 Is the inhalation of low-dose CO a prohibited method for elite athletes?

Before focusing on the physiological effects and on potential adverse effects of CO inhalation it is important to clarify that the current prohibited list of the World-Anti-Doping-Agency (WADA) could be interpreted in a way that the inhalation of CO to increase red blood cell mass and thereby performance is already prohibited at all times under “*M1. Manipulation of Blood and Blood Components*” (https://www.wada-ama.org/en/resources/world-anti-doping-code-and-international-standards/prohibited-list). This would obviously not apply to the use of CO as a tracer in diagnostic procedures such as tHb-mass- or lung diffusion capacity measurements.

## 3 Effects of low-dose CO inhalation on total hemoglobin mass and exercise performance

Studies investigating the effects of low-dose CO on tHb-mass and exercise performance have yielded mixed results. Acute low-dose CO administration has been shown to reduce oxygen-carrying capacity and decrease maximal oxygen uptake (VO_2max_) as CO occupies O_2_ binding sites on Hb and prevents Hb from fulfilling its role in oxygen transport (Schmidt and Prommer, 2005; Klausen et al., 1983). However, these effects are transient, with CO typically cleared from the system within 12 h (Schmidt and Prommer, 2005). Recent research has shown that acute low-dose CO does not affect exercise economy (i.e., oxygen consumption during submaximal exercise) in recreationally active men (Kane et al., 2016). In addition, single daily low-dose CO inhalation (1.2 mL/kg body weight) over a 12-day period did not significantly affect tHb-mass, VO_2max_, lactate threshold, economy, or peak power output in moderately trained men (VO_2max_ 49 mL/min/kg) (Ryan et al., 2016).

In contrast, Wang et al. reported that inhaling a CO bolus (1 mL/kg body mass) five times a week prior to treadmill training increased tHb-mass by 3.7% in trained soccer players (VO_2max_ 58 mL/min/kg), although a 2.8% increase was also observed in the control group (Wang et al., 2019). Pecorella et al. (2015) reported no improvement in single-leg VO_2max_ after CO inhalation (1 h/day for 5 days, 200 ppm) in healthy subjects, although increases in muscle mitochondrial density, myoglobin content and glucose transporter (GLUT 4) were found (Pecorella et al., 2015).

In addition to these findings, Schmidt et al. (2020) showed that chronic intermittent low-dose CO inhalation of moderately trained athletes (VO_2max_ 56 mL/min/kg), aimed at continuously increasing COHb concentration in the blood by about 5% over a period of 3 weeks, resulted in a 4.8% increase in tHb-mass (comparable to the effects of altitude training). After 1 week, EPO levels tended to increase, as did reticulocytes and the immature reticulocyte fraction. EPO levels were suppressed after CO administration ceased. Furthermore, CO administration shifted the oxygen dissociation curve to the left, with possible effects on oxygen unloading and tissue oxygenation. In addition to physiological adaptations, a relationship between individual changes in tHb-mass and VO_2max_ was reported, with an increase in VO_2max_ of approximately 4 mL/min per 1 g increase in tHb-mass, although VO_2max_ was not significantly increased. Furthermore, a recent study found that the addition of twice-daily CO inhalation (increasing CO levels to ∼10%) to a live-high and train-high (LHTH) training camp (3 weeks at 2,100 m) induced larger increases in tHb-mass compared to LHTH without CO breathing in male cyclist (VO_2max_ > 70 mL/min/kg) (Urianstad et al., 2024). Although improvements in endurance performance (i.e., maximal power output during incremental exercise testing, power output at lactate threshold, and VO_2max_) were found compared to a sea level control group, performance improvements were similar between the LHTH training group with and without additional CO inhalation (Urianstad et al., 2024). It should be noted that there is a scientific debate as to whether tHb-mass is increased (Robach and Lundby, 2012; Millet et al., 2019) and endurance performance at sea level improved after altitude training in elite athletes (Siebenmann and Dempsey, 2020; Millet and Brocherie, 2020).

Overall, the limited number of studies with participants of varying fitness level, from recreationally active to highly-trained, and the inconsistent study results do not allow a definitive conclusion to be drawn. Methodological issues also need to be considered. For example, the lack of statistical indicators (i.e., lack of information on main- and interaction effects), as in the study by Wang et al. (2019), could call into question the true effect of the application, at least when combined with exercise. For application in elite endurance athletes, it should also be mentioned that the absolute training intensity, especially in high-intensity sessions, might be impaired by increased COHb concentrations (due to reductions in VO_2max_ and time to exhaustion (Schmidt and Prommer, 2005; Ekblom and Huot, 1972; Hogan et al., 1990; Richardson et al., 2002) and increased heart rate, lactate concentration and perceived exertion during submaximal exercise (Kane et al., 2016)) and thereby the quality of training may suffer from the hypoxaemic state.

## 4 Potential health concerns and other considerations

While high doses of CO are undeniably lethal (Smollin and Olson, 2010), the safety of low-dose exposure remains a subject of debate. Various health effects associated with CO exposure, ranging from mild cardiovascular and neurobehavioral effects to severe consequences (Schmidt et al., 2020), warrant careful consideration. The following concerns merit further investigation:

a. Individual variability: Symptoms, signs and prognosis of acute CO poisoning often vary among individuals and do not consistently correlate with COHb levels (Bansal et al., 2024; Schmidt et al., 2020; Raub et al., 2000).
b. CO poisoning: CO poisoning includes both, tissue hypoxia and direct cellular changes through various mechanisms, e.g., mitochondrial inhibition, free radical generation, apoptosis, immune-mediated injury, inflammatory effects (Hampson et al., 2012; Rose et al., 2017).
c. Short and long-term adverse health effects: Numerous human clinical trials have concluded that CO levels up to 6.4% COHb can be considered safe (Bansal et al., 2024). During CO-rebreathing for tHb-mass determination COHb levels of up to 10% have been reported (Siebenmann et al., 2017); for comparison regular smokers have COHb levels of around 3–8% and heavy smokers may reach levels >20% (Bansal et al., 2024; Hess, 2017). Depending on the COHb level, adverse symptoms include headache, dizziness, fatigue, nausea/vomiting, altered mental status, chest pain, shortness of breath, loss of consciousness and death (Rose et al., 2017; Hess, 2017; Weaver, 2009). Mild CO intoxication symptoms (e.g., headache, fatigue) may occur at COHb concentrations >10%, with severe symptoms manifesting at COHb >20–25% (Siebenmann et al., 2017). Severe CO poisoning can result in brain (e.g., cognitive dysfunction, neurological deficits, neuronal necrosis and apoptosis) and myocardial injury (Rose et al., 2017). Currently, it is not possible to establish a safe upper limit for continuous intermittent CO use due to limited long-term studies (Schmidt et al., 2020).
d. Occupational exposure limits: While workers are permitted exposure to 35–50 ppm of CO for a 40-h work week (increasing COHb to about 5%), these limits are designed for occupational safety and may not account for potential effects of long-term exposure or use for performance enhancement (Schmidt et al., 2020; Hess, 2017).
e. Oxygen dissociation curve shift: CO administration shifts the oxygen dissociation curve to the left (Hogan et al., 1990), potentially hindering oxygen unloading and tissue oxygenation (Schmidt et al., 2020). However, the relatively short half-life of COHb suggests only a transient hypoxemia effect. Conversely, prolonged administration may result in a rightward shift due to reduced mean erythrocyte age caused by erythropoiesis (Schmidt et al., 1987), though this hypothesis requires further validation.
f. Potential for misuse: There is a risk that athletes might abuse CO, potentially resulting in serious health problems or death (Schmidt et al., 2020).

## 5 Conclusion

The measurement of tHb by the CO rebreathing technique is a well-established method in exercise science (Siebenmann et al., 2017) and training practice. Single measurements seem not to influence performance (Ryan et al., 2016). It should be noted that the blood CO concentration at the time of measurement can vary between individuals due to different life habits (including smoking) and environmental exposures (e.g., urban pollution). The limited research on low-dose (chronic intermittent) CO exposure suggests stimulated erythropoiesis, which may have potential performance-enhancing effects in athletes by increasing the number of circulating erythrocytes and oxygen transport capacity (evidence low to moderate) (Schmidt et al., 2020). However, this finding raises complex ethical issues and significant health concerns when used by elite athletes. With the exception for diagnostic application, the inhalation of carbon monoxide can be interpreted as a prohibited method by WADA. Therefore, further studies in elite athletes to gain more information on its potential performance enhancing effects may be scientifically interesting but could be viewed critically if endurance performance improvements are confirmed.
